# Parent-young communication on sexual and reproductive health issues and its association with sex and perceptions of young people in Ethiopia, 2020: a systematic review and meta-analysis

**DOI:** 10.1186/s13690-020-00515-x

**Published:** 2020-12-14

**Authors:** Melaku Yalew, Bezawit Adane, Bereket Kefale, Yitayish Damtie, Mastewal Arefaynie, Shambel Wedajo, Abebayehu Bitew, Yitbarek Wasihun

**Affiliations:** 1 Department of Reproductive and Family Health, School of Public Health, College of Medicine and Health Sciences, Wollo University, Dessie, Ethiopia; 2 Department of Epidemiology and Biostatistics, School of Public Health, College of Medicine and Health Sciences, Wollo University, Dessie, Ethiopia; 3 Department of Epidemiology and Biostatistics, School of Public Health, College of Medicine and Health Sciences, Bahir Dar University, Bahir Dar, Ethiopia; 4 Department of Health Promotion, School of Public Health, College of Medicine and Health Sciences, Wollo University, Dessie, Ethiopia

**Keywords:** Parent-young, Discussion, Communication, Meta-analysis, Ethiopia

## Abstract

**Background:**

There is no national representative and conclusive data regarding parent-young communication. Therefore, this study aimed to estimate the pooled prevalence of parent-young communication on sexual and reproductive health issues and its association with sex and perceptions of young people about its importance in Ethiopia, 2020.

**Methods:**

The Preferred Reporting Items for Systematic Review and Meta-analysis (PRISMA) was used during systematic review and meta-analysis. The study included both published and gray literatures which were searched using appropriate key terms. The articles were searched from different databases: PubMed, Cochrane Library, PsycINFO, CINAHL, Global Health, HINARI and Google scholar. Data were extracted in a Microsoft Excel sheet and STATA/SE 14 was used for meta-analysis. I^**2**^ and Egger test statistics were used to test heterogeneity and publication bias respectively.

**Results:**

Twenty-nine articles were included in this systematic review and meta-analysis. The pooled prevalence of parent-young communication on sexual and reproductive health issues in Ethiopia was 42.96% [95% CI: (36.91, 49.02)]. Positive perception of young people towards parent-young communication [AOR = 3.72, 95% CI: (2.87, 4.86)] and female sex [AOR = 1.62, 95% CI: (1.12, 2.34)] were significantly associated with parent-young communication on sexual and reproductive health issues.

**Conclusions:**

The prevalence of parent-young communication on sexual and reproductive health issues in Ethiopia was low as compared to other literatures conducted outside Ethiopia. Both perceptions of young people towards parent-young communication and sex were significantly associated with parent-young communication on sexual and reproductive health issues. Interventions targeting males and young who had negative perceptions regarding parent-young communication should be the primary focus of the government and their parents.

**Review registration:**

The protocol of this systematic review and meta-analysis was registered in the PROSPERO International prospective of systematic reviews with a specific registration number: CRD42020161252.

## Background

According to the World Health Organization (WHO), young were populations aged from 10 to 24 years. Globally, the numbers of the young population were estimated to be 1.8 billion, of which 90% of them were lived in developing countries [1, 2]. Adolescent is the time of experimentation and in the course of such experimentation, they are often encountered in high-risk situations especially Human Immune Deficiency Virus (HIV) and unwanted pregnancy [3, 4]. The prevalence of parent-adolescent communication on reproductive health in Bangladesh was 65.6% [5] and ranged between 21 to 46.7% in Nigeria [6, 7]. Whereas, it was 53% in Kenya [8] and 60% in Zambia [9]. In Ethiopia, the prevalence of parent-young communication on sexual and reproductive health issues was ranged from 8.84 to 82.7% [10, 11].

The problem in parent-adolescent communication has been associate with an increase in a wide range of adolescent risk behaviors [8, 12–16]. Evidence showed that 29% of premarital sex was caused by improper parenting style [17]. Good parent-adolescent communication enables them to know their HIV status which would have an impact on HIV transmission [18]. Parental communication had an impact on the mental wellbeing of adolescents [19]. The other advantage of good parental communication was that it can delay early sexual initiation [20, 21].

Parent-young communication was associated with socio-demographic characteristics of young (age, sex, educational status) [9, 15, 22], parental characteristics (educational status and marital status) [5, 23, 24]. Improving sexual and reproductive health of young is one of the priorities of Sustainable Developmental Goals SDGs) [25]. In line with this, Ethiopia also develops strategies particularly for adolescents and youth which would apply to the end of 2020 [26]. Parent-young communication on sexual and reproductive health issues was a little bit investigated and studied in different parts of Ethiopia [27–35]. But, the finding of each study was highly varied and there is no single national representative estimate regarding it [11, 27–29, 36–39]. So, this study aimed to estimate the pooled prevalence of parent-young communication on sexual and reproductive health issues and its association with sex and perceptions of young people about its importance in Ethiopia, 2020.

## Methods

### Study design, search strategy and registration

The study was designed based on the Preferred Reporting Items for Systematic Reviews and Meta-Analysis Protocols (PRISMA-2009 Guidelines) [40]. It was conducted using published and unpublished articles and the articles were searched from different databases: (PubMed, Cochrane library, PsycINFO, CINAHL, Global Health, HINARI and Google scholar). All potential articles were accessed by using a combination of keywords/indices like; “prevalence”, “magnitude”, “parent adolescent”, “parent youth”, “parent young”, “communication”, “open communication”, “discussion”, “determinants”, “factors associated”, “associated factors”, “predictors”, “risk factors”, “sexual”, “sexuality”, “sexual issue”, “reproductive health” which were developed according to Medical Subject Headings (MeSH). All these key terms were searched by a combination of Boolean operators “AND” or “OR” as appropriate and the search was done by two authors independently (MY and BA). The protocol of this systematic review and meta-analysis was registered in the PROSPERO International prospective of systematic reviews with a specific registration number: **CRD42020161252**.

### Study selection and eligibility criteria

#### Inclusion criteria

- ❖ Population: This systematic review included studies conducted among young and/or parents having young (aged 10 to 24 years) in Ethiopia.
- ❖ Exposure: Female young and young who had positive perception towards parent-young communication.
- ❖ Comparison: Male young and young who had negative perception towards parent-young communication.
- ❖ Outcome: Studies conducted parent-young communication as primary outcome.
- ❖ Study design: All observational studies were included
- ❖ Time frame: All studies irrespective of data collection and publication year until the end of November 1, 2020
- ❖ Publication: Either published in peer-reviewed journals or unpublished studies.
- ❖ Language: studies published only in English language were included in this systematic review and meta-analysis.

#### Exclusion criteria

- ❖ Studies in which the outcome did not clearly reported
- ❖ Studies in which the full texts were not available after 2 times authors request were excluded from systematic review and meta-analysis.

#### Variable measurements

Parent-young communication on sexual and reproductive health issues was measured in two ways. Thirteen articles used as having talked about at least one [11, 28, 30, 32, 34, 36, 38, 41–46] and the rest 16 articles were used at least two [10, 27, 29, 31, 33, 35, 37, 39, 47–54] of the following sexual and reproductive health related topics with their parents with in the past 6 months: a) body change during puberty, b) menstruation, c) abstinence, d) condom use and where to get condom, f) relationship with opposite sex, g) STI including HIV/AIDS, h) family planning, i) abortion, j) unplanned pregnancy and k) sexual organs.

#### Study quality appraisal and data extraction

Those articles identified in all databases were exported to Endnote X8 and duplicate files were excluded. The remaining articles and abstracts were independently screened by two groups (YD and BK) for inclusion in the full-text appraisal. It was assessed using Joanna Brigg’s Institute (JBI) critical appraisal checklist according to the study design of each article [55, 56]. Two independent authors (SW and AB) assessed the quality of the articles and the differences in the scales result was settled by taking the average result of both reviewers.

Data were extracted using Microsoft excel 2010 sheet and the sheet contained the following list of variables for the first objective: authors name followed by initials, year of study, year of publication, study setting, study design, sample size, response rate, quality score, sex of participants, region, study finding. For the second objective, in addition to the above mentioned, studies reported at least one of the above mentioned factors as predictors were identified and extracted. Two authors (MY and YW) extract the data for both objectives and any disagreements between the two authors during extractions were solved through discussion and consensus.

#### Data synthesis and statistical analysis

The data extracted in the Microsoft Excel sheet format was exported into STATA/SE 14 version statistical software for further analysis. The pooled effect of the point estimate of parent-young communication in Ethiopia was calculated by DerSimonian & Liard’s method of random effect model at *P*-value less than 0.05 [57]. Statistical significance for heterogeneity with I^2^ tests greater than 75% was taken as high heterogeneity and it was subjected to sub-group and sensitivity analysis. Finally, publication bias was assessed by using Egger’s weighted regression test method (*p*-value < 0.05) which was considered as statistically significant publication bias [58].

## Results

### Study selection

**Fig. 1:**
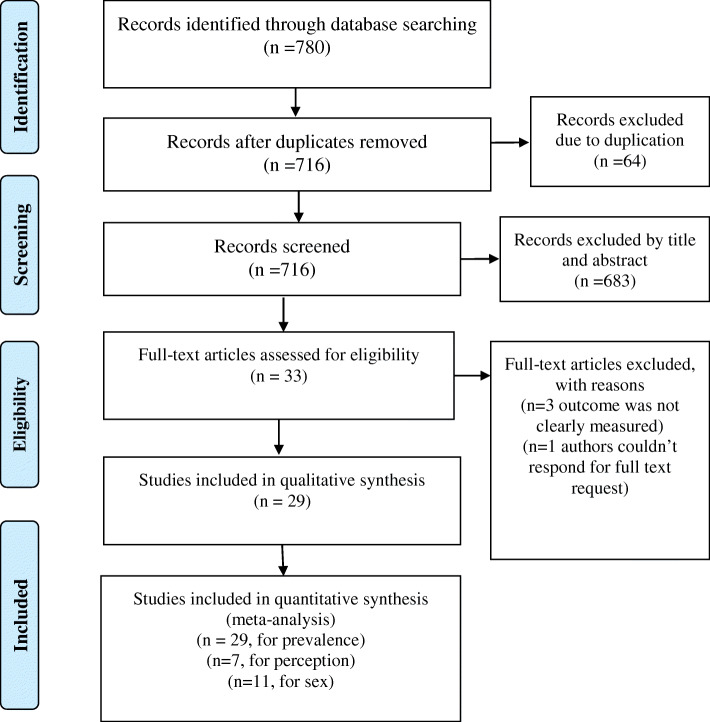
PRISMA flow diagram of parent-young communication on sexual and reproductive health issues and its association with sex and perceptions of young people, a systematic review and meta-analysis, Ethiopia, 2020

The review found a total of 780 articles. Sixty-four of them were removed due to duplication and 683 records also excluded after screening by title and abstract. The remaining 33 of them were screened for eligibility and appraised based on JBI checklist. Again 4 of them were excluded due to reason (outcome was not clearly reported or the authors couldn’t respond for full text request). A total of 29 full-text articles were included in systematic review and meta-analysis (Fig. 1).

### Descriptive characteristics of included studies

All included articles were cross-sectional studies and the minimum and the maximum sample size was 262 and 2075 participants in a study conducted in Shambo and Nekemte town, Oromia respectively [43, 46]. A total of 16,721 young and/or parents had young were included and nearly half (48.28%) of the studies were from both preparatory and high school students [10, 30, 36, 38, 41, 42, 44, 45, 47–52] and six of them were community based [11, 28, 43, 46, 53, 54] (Table 1).

**Table 1:** Characteristics of studies included for parent-young communication on sexual and reproductive health issues and its association with sex and perceptions of young people, a systematic review and meta-analysis, Ethiopia, 2020

Authors	Study year	Year of Publication	Regions	Study setting	Sample size	Prevalence	RR%	Quality score
Ayalew M et al.	2011	2014	Harari	High	641	36.82	92.0	7
Ayehu A et al.	2014	2016	Amhara	Community	746	51.74	95.5	7
Beyene Det al	2015	.	Oromia	Both	640	44.84	98.6	7
Chane T et al.	2017	2018	Amhara	Both	323	82.66	97.3	5
Fanta M et al.	2015	2017	SNNP	Both	740	40.68	95.8	7
Feyisa M et al.	2017	.	Oromia	Both	378	62.43	96.0	6
Fikre M el al	2009	.	SNNP	High	674	30.42	97.1	7
Habte N et al.	2017	2019	Oromia	Both	394	47.21	100	5
Kinfe M et al.	2016	2018	SNNP	Preparatory	550	36.18	97.3	6
Kusheta S et al.	2016	2019	SNNP	Both	411	35.04	96.0	6
Leul S et al.	2001	.	Addis Ababa	Preparatory	378	34.39	96.2	5
Masresha S et al	2019	.	Amhara	Both	359	56.27	97.6	5
Mekie M et al.	2019	2019	Amhara	Preparatory	394	68.53	100	6
Mekonnen M et al.	2016	2017	Amhara	High	674	30.42	97.3	7
Melaku Y et al.	2012	2014	Tigray	High	807	43.49	100	7
Mengistu T et al	2012	.	Amhara	Both	688	50.58	98.9	7
Neme D et al	2016	2020	Oromia	Both	292	55.82	100	5
Shiferaw K et al.	2012	2014	Amhara	Both	688	36.92	98.9	7
Shwasinad S et al.	2016	2017	SNNP	Both	356	28.93	100	5
Tadelle M et al.	2017	2018	Amhara	Preparatory	394	28.93	100	6
Tesso D et al.	2011	2012	Oromia	Community	2075	42.51	.	8
Yesus D et al.	2006	2010	Benishagul	Both	412	28.88	97.6	6
Yohannes Z et al	2014	2015	Tigray	Both	521	57.58	97.0	7
Yohannes Z et al.	2015	2015	SNNPR	Both	660	59.10	96.5	7
Yowhanes Z et al.	2013	2016	Tigray	High	521	57.58	97.0	7
Assebe T et al.	2009		Oromia	community	262	38.17	100	5
Wolde T et al.	2018	2019	Amhara	community	645	8.84	100	7
Bekele D et al	2019		Oromia	community	347	23.05	100	5
Yadeta T et al.	2010	2014	Harari	community	751	28.76	97.8	6

Benishangul-Benishangul Gumze, Both-both preparatory and high school, RR-Response Rate and SNNP-Southern Nation Nationalities and Peoples Representative

### Prevalence of parent-young communication on sexual and reproductive health issues

**Fig. 2:**
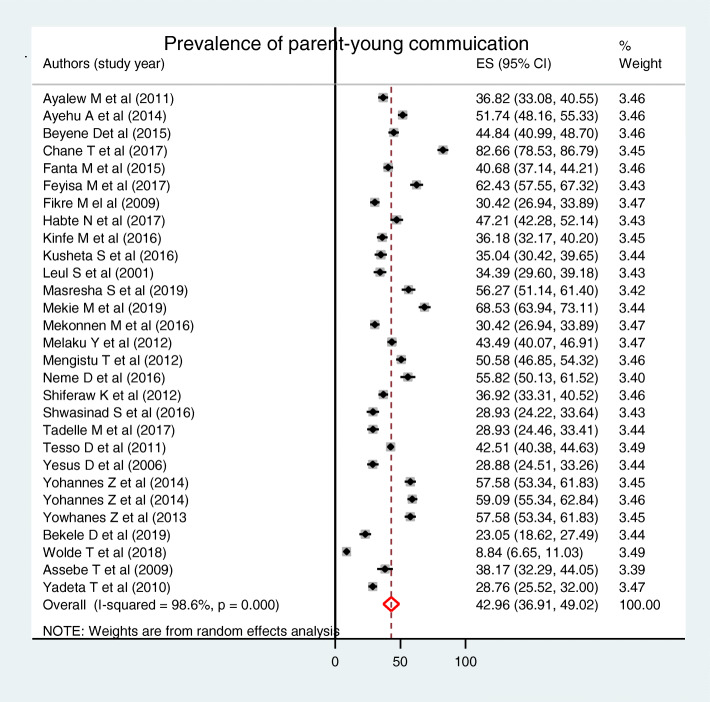
Forest plot of the pooled prevalence of parent-young communication on sexual and reproductive health issues, a systematic review and meta-analysis, Ethiopia, 2020

**Fig. 3:**
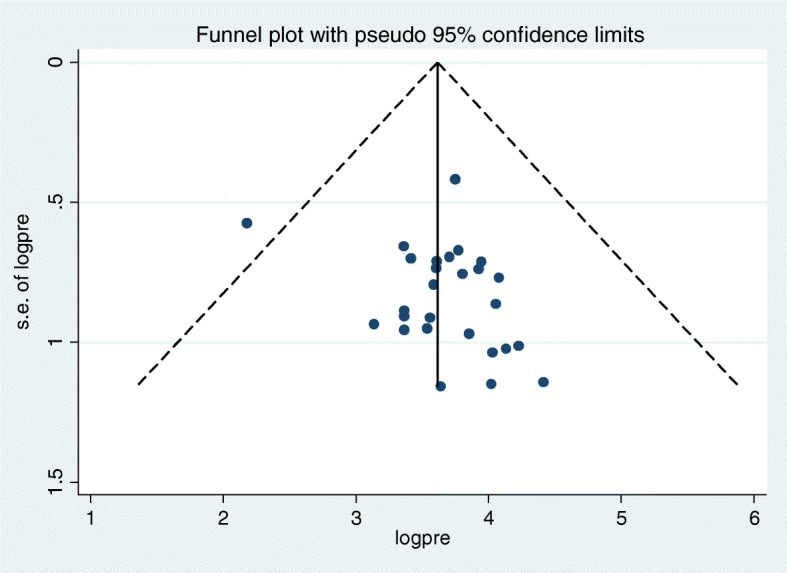
Funnel plot showing publication bias of parent-young communication on sexual and reproductive health issues and its association with sex and perceptions of young people, a systematic review and meta-analysis, Ethiopia, 2020

Parent-young communication on sexual and reproductive health issues was ranged between 8.84 to 82.67% in unpooled estimate [10, 52]. The pooled prevalence of parent-young communication on sexual and reproductive health issues was 42.96% [95% CI: (36.91, 49.02)]. As it was illustrated in the figure, there was considerable heterogeneity (I^2^ = 98.6%, *P* value = 0.0001) among studies included in the analysis (Fig. 2). It was also 45.05% [95% CI: (34.97, 55.14)] and 41.27% [95% CI: (33.69, 48.85)] for those young who talked one and two sexual and reproductive health issues respectively. The sensitivity analysis indicated that there was no single influential estimate that could attributed to source of heterogeneity. The funnel plot of pooled prevalence was symmetrical (Fig. 3) and the Egger test revealed that there was no statistical evidence of publication bias (*P* = 0.112).

### Subgroup analysis

**Fig. 4:**
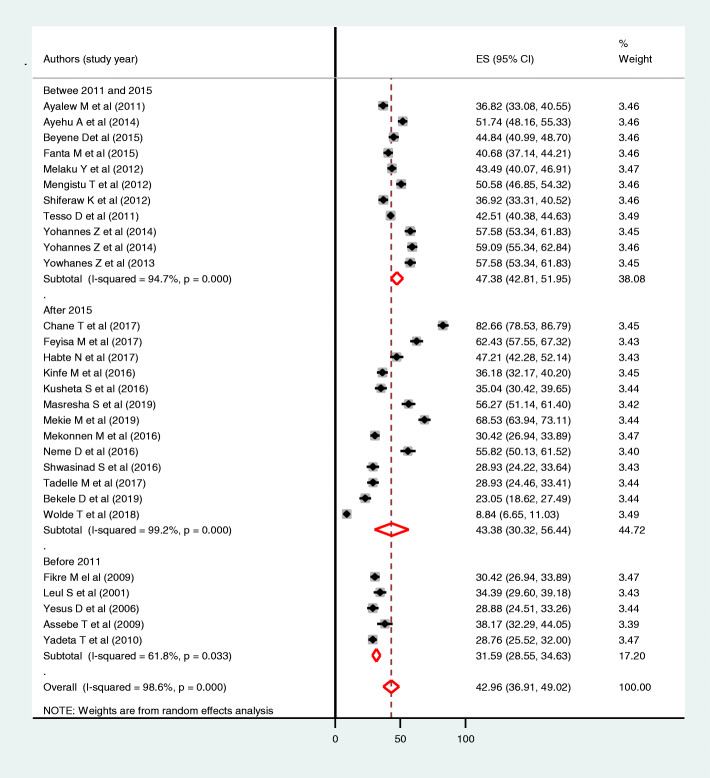
Forest plot of subgroup analysis for prevalence of parent-young communication on sexual and reproductive health issues by study year using the random effect model, a systematic review and meta-analysis, Ethiopia, 2020

Subgroup analysis was done by different parameters (region, study setting, study year, response rate and type of participants) to observe the possible sources of heterogeneity. Despite heterogeneity was not totally resolved in subgroup analysis, those studies conducted before 2011 had moderate heterogeneity (I^2^ = 61.8%, *P* value = 0.033) (Fig. 4). The highest and the lowest prevalence of parent-young communication were observed in Tigray and Benishangul Gumz respectively [52.82, 95% CI: (43.02, 62.61] and [28.88, 95% CI: (24.51, 33.26)]. Similarly, the prevalence of parent-young communication was significantly different among young [45.89, 95% CI: (40.76, 51.03)] and parents [24.56, 95% CI: (11.55, 37.57)].

### Factors associated with parent-young communication

**Fig. 5:**
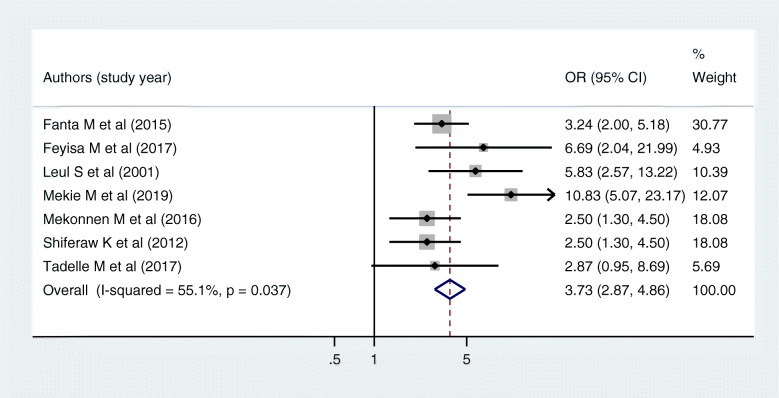
Forest plot of the pooled estimate of the effect of perceptions of young people on parent-young communication on sexual and reproductive health issues, a systematic review and meta-analysis, Ethiopia, 2020

The effect of perceptions of young people regarding parent-young communication on sexual and reproductive health issues was estimated by using seven articles. From those, only one of them was not significant [39] and the others were positively significant [31, 33, 35, 41, 47, 51]. The pooled odds of parent-young communication on sexual and reproductive health issues among young who had positive perception regarding parent-young communication was increased by 4 as compared to counterparts [AOR = 3.72, 95% CI: (2.87, 4.86)] (Fig. 5). The egger test showed that there was no statistical evidence of publication bias (*P* value = 0.334).

**Fig. 6:**
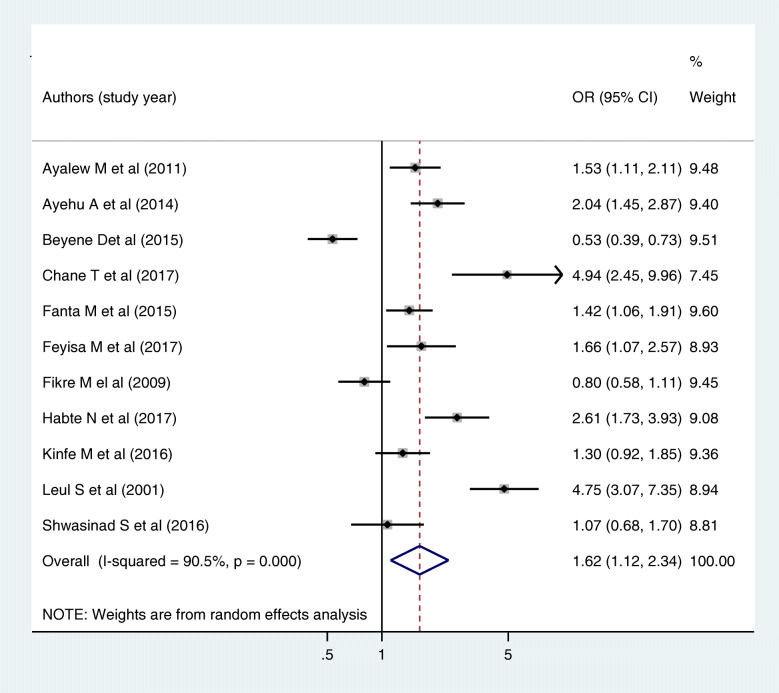
Forest plot of the pooled estimate of the effect of sex on parent-young communication on sexual and reproductive health issues, a systematic review and meta-analysis, Ethiopia, 2020

The effect of sex on parent-young communication on sexual and reproductive health issues was estimated by using 11 articles. From those, only one of them was negatively significant [36], three of them were not significant [29, 37, 38] and the rest were positively significant [10, 27, 28, 35, 41, 47, 48]. Using the random effect model, the pooled odds of parent-young communication on sexual and reproductive health issue among females was 1.6 times high as compared to males [AOR = 1.62, 95% CI: (1.12, 2.34)] (I^2^ = 90.5%, *P* value = 0.000) (Fig. 6). The egger test shown that there was no statistical evidence of publication bias (*P* value = 0.073).

## Discussion

The pooled prevalence of parent young communication on sexual and reproductive health issue in Ethiopia was 42.96% [95% CI: (36.91, 49.02)]. The pooled prevalence of parent-young communication was in line to a study conducted in Nigeria (46.7%) [6]. But, the result of this study was low as compared to a study conducted in Kenya (53%) [8] and Zambia (60%) [9]. Again the finding of this study was low as compared to a study in Bangladesh (65.6%) [5]. The possible explanation for this discrepancy may be due to socio-cultural difference related to parenting style [22]. In addition, traditional norms and religious beliefs of the countries may be another source of variation [24]. The highest and the lowest prevalence of parent young discussion were observed in Tigray and Benishangul Gumz respectively [52.82, 95% CI: (43.02, 62.61] and [28.88, 95% CI: (24.51, 33.26)]. The possible reason for this visible difference may be due to that Tigray region was relatively developed as compared to Benishangul Gumz. Moreover, the participants in Tigray region may be high possibility to have media exposure than that of Benishangul Gumz.

In addition, the highest prevalence of parent-young communication was observed in studies conducted among young as compared to studies conducted among parents. This may be due to the fact that parents had less exposure to education and information than young. Moreover, the parents might think it as a positive reinforcing factor for early sexual initiation.

The pooled odds of parent-young communication on sexual and reproductive health issues among young who had positive perception regarding parent-young communication were statistically high as compared to counterparts. The finding of this study was in agreement with a study conducted in Bangladesh [5]. It is also congruent with a study conducted in Myanmar [23]. The possible reason for this association may be their perception was highly affected by the culture what they were growing and sometimes they may also perceived as their parents have negative perception regarding parent-young communication [17].

The pooled odds of parent-young communication on sexual and reproductive health issues among females were high as compared to males. The finding was congruent to a study conducted in Nigeria [6]. The finding was also in agreement with a study conducted in Rwanda [15]. The possible reason for this association may be due to the fact that males were feeling ashamed and it is culturally unacceptable to talk about sexual matters. Even, they may wrongly perceived it as it would be interpreted as signs of sexual initiation [59]. Moreover, in the context of Ethiopia, females were wasting most of their times in the household which may increase the contact and possibilities of discussion than males.

Despite its strength, the study was not without limitation. The review was restricted to articles published in the English language and it may not be representative for articles published in other languages. Not only this, but also all the studies included in meta-analysis were cross-sectional and the pooled estimate may be influenced by study design.

## Conclusions

The prevalence of parent-young communication on sexual and reproductive health issue in Ethiopia was low as compared to other literatures. Both positive perceptions of young people towards parent-young communication and female sex were significantly associated with parent-young communication on sexual and reproductive health issues. So, the government should focus on interventions that could change the perception of young on parent-young discussion. Interventions targeting male and young who had negative perception regarding parent-young communication should be the primary focus of the parents.

## Data Availability

All the data used and analyzed during this study are available from the corresponding author on reasonable request.

## Abbreviations

AIDS: Acquired Immune Deficiency Syndrome
HIV: Human Immune Deficiency Virus
JBI: Joanna Brigg’s Institute
WHO: World Health Organization
